# TAB1-Induced Autoactivation of p38α Mitogen-Activated Protein Kinase Is Crucially Dependent on Threonine 185

**DOI:** 10.1128/MCB.00409-17

**Published:** 2018-02-12

**Authors:** Dibesh Thapa, Charlie Nichols, Rekha Bassi, Eva Denise Martin, Sharwari Verma, Maria R. Conte, Vittorio De Santis, Gian F. De Nicola, Michael S. Marber

**Affiliations:** a British Heart Foundation Centre of Excellence, Department of Cardiology, The Rayne Institute, St Thomas' Hospital, King's College London, London, United Kingdom; b Randall Division, King's College London, London, United Kingdom

**Keywords:** mitogen-activated protein kinases, p38α, autophosphorylation, TAB1, scaffold proteins, myocardial ischemia, autophosphorylation, p38 alpha kinase

## Abstract

p38α mitogen-activated protein kinase is essential to cellular homeostasis. Two principal mechanisms to activate p38α exist. The first relies on dedicated dual-specificity kinases such as mitogen-activated protein kinase kinase (MAP2K) 3 (MKK3) or 6 (MKK6), which activate p38α by phosphorylating Thr180 and Tyr182 within the activation segment. The second is by autophosphorylation of Thr180 and Tyr182 in *cis*, mediated by p38α binding the scaffold protein TAB1. The second mechanism occurs during myocardial ischemia, where it aggravates myocardial infarction. Based on the crystal structure of the p38α-TAB1 complex we replaced threonine 185 of p38α with glycine (T185G) to prevent an intramolecular hydrogen bond with Asp150 from being formed. This mutation did not interfere with TAB1 binding to p38α. However, it disrupted the consequent long-range effect of this binding event on the distal activation segment, releasing the constraint on Thr180 that oriented its hydroxyl for phosphotransfer. Based on assays performed *in vitro* and *in vivo*, the autoactivation of p38α(T185G) was disabled, while its ability to be activated by upstream MAP2Ks and to phosphorylate downstream substrates remained intact. Furthermore, myocardial cells expressing p38α(T185G) were resistant to injury. These findings reveal a mechanism to selectively disable p38α autoactivation and its consequences, which may ultimately circumvent the toxicity associated with strategies that inhibit p38α kinase activity under all circumstances, such as with ATP-competitive inhibitors.

## INTRODUCTION

Protein p38α is a stress-activated serine/threonine kinase that relays extracellular signals to coordinate cellular responses. p38α is ubiquitously expressed, involved in numerous signaling networks and essential for cellular homeostasis, development and survival (1–3). The kinase activity of p38α is controlled by the phosphorylation of two key residues within the activation segment, Thr180 and Tyr182. Typically, these events are executed in *trans* by dual-specificity upstream kinases, mitogen-activated protein kinase kinase (MAP2K) 3 (MKK3) or 6 (MKK6) (4). However, under certain circumstances, p38α can autophosphorylate these key residues in *cis*, an event triggered by binding to a scaffold protein, transforming growth factor β-activated protein kinase binding protein 1 (TAB1) (5, 6). This atypical activation mechanism does not depend on archetypal upstream MAP2Ks and is relevant to myocardial infarction, where it seems to aggravate lethal injury of the heart (7–11). Identification of the pathophysiological processes in which p38 is involved has benefitted from the early availability of several ATP-competitive inhibitors. However, despite the development of a variety of drugs, clinical trials to date have failed due to toxicity or dosages that likely lie below the therapeutic range in order to avoid toxicity (12). Toxicity is almost certainly an “on-target” effect since it occurs with diverse scaffolds binding to different locations in or around the ATP-binding pocket. The occurrence of toxicity is not surprising considering the ubiquitous nature of p38α expression and its involvement in signaling networks that maintain normal cell function. For example, in cardiomyocytes p38α itself plays a beneficial role in promoting protection in preconditioning but is harmful in myocardial ischemia (13, 14). Targeting TAB1 would be futile, as its knockout causes embryonic lethality and it plays an important role in the TAK1 signaling pathway (15, 16). As a result, selectively targeting the TAB1-dependent autoactivation of p38α may provide an opportunity to avoid the toxicity seen with complete inhibition of p38α kinase activity, while still deriving therapeutic benefit.

**FIG 1:**
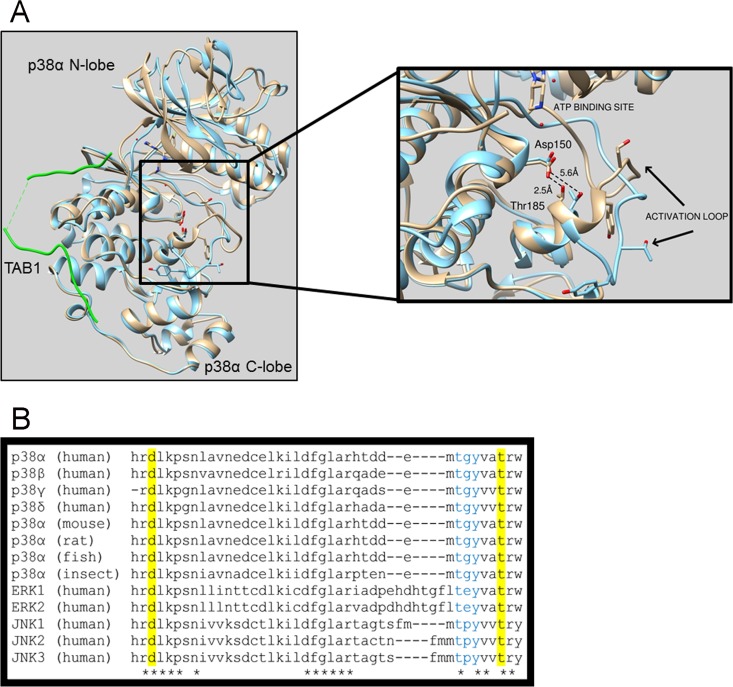
(A) Superimposition of the crystal structures of free wtp38α (blue) and wtp38α (gray) in complex with TAB1 (green). In the free structure the side chain oxygens of Thr185 and Asp150 are separated by 5.6 Å, which prevents the formation of a hydrogen bond, and the activation loop is facing away from the catalytic site, a position not compatible with the autophosphorylation reaction. Upon TAB1 binding, Tyr182 to Thr185 form an alpha-helical segment that is stabilized by the hydrogen bond formed between Thr185 and Asp150 with side chain oxygens now 2.5 Å apart, typical of hydrogen bond formation. Consequently, the activation loop moves toward the catalytic site, bringing the T-G-Y motif in proximity to the γ-phosphate position of ATP, within its binding site, a position compatible with the autophosphorylation reaction. (B) Asp150 and Thr185 are conserved across p38 isoforms, species, and other mitogen-activated protein kinases.

To target the interaction between p38α and TAB1, we need to understand the structural detail of the p38α-TAB1 complex at an atomic level. Therefore, we previously solved the crystal structure of p38α and the interacting region of TAB1, which showed that long-range structural changes within p38α result in an increased affinity for ATP (6). The N-terminal and C-terminal lobes of p38α move toward each other, and this movement positions the activation loop containing Thr180 and Tyr182 toward the catalytic site, which facilitates autophosphorylation. TAB1 binding also causes residues Tyr182 to Thr185 to extend into a short α-helical segment, accompanied by hydrogen bond formation between the side chains of Thr185 in the activation segment and Asp150 of the HRD motif (Fig. 1A). This hydrogen bond holds the activation loop in its new position where Thr180 and Tyr182 lie close to the catalytic site, likely promoting the autophosphorylation reaction. In keeping with these observations, Thr185 and Asp150 are conserved across p38 isoforms and species (Fig. 1B).

In this study, we hypothesized that preventing the formation of the hydrogen bond between Thr185 and Asp150 will interfere with the autophosphorylation of Thr180 and Tyr182, since the required and unique orientation of the activation segment will become energetically unfavorable. To investigate our hypothesis, we mutated Thr185, since Asp150 lies within the HRD motif, which is involved in substrate recognition and forms part of a central spline conserved among all protein kinases (17).

## RESULTS

### TAB1 still binds p38α(T185G).

p38α autoactivation is caused by TAB1 interacting with p38α; i.e., without the p38α-TAB1 interaction, the autophosphorylation of p38α's T-G-Y motif is not promoted. Before testing our hypothesis that the mutation in p38α(T185G) impairs TAB1-induced p38α autoactivation, we needed to confirm that the p38α(T185G) mutant kinase is able to bind TAB1. Using isothermal titration calorimetry (ITC), we found that p38α(T185G) binds to TAB1 with an affinity similar to that of wild-type p38α (wtp38α) (Table 1). In our biophysical and crystallographic experiments, we have used a peptide spanning residues 384 to 412 of TAB1 [TAB1(384–412)], and we have previously shown that no other residue beyond this region is involved in the interaction with p38α and that the peptide can recapitulate the behavior of full-length TAB1 *in vivo* (6).

**TABLE 1:** Thermodynamic parameters, determined by ITC, for the association of wtp38α with TAB1(384–412) and of p38α(T185G) with TAB1(384–412)^*a*^

Complex	*n*	*K_d_* (μM)	Δ*G* (kcal/mol)	Δ*H* (kcal/mol)	−*TΔS* (kcal/mol)
wtp38α-TAB1(384–412)	0.8	2.9	−7.5	−13.6	6.1
p38α(T185G)-TAB1(384–412)	0.7	1.1	−8.1	−9.6	1.5

a The data show that p38α(T185G) is able to bind to TAB1 with thermodynamic characteristics that are similar to those for wtp38α, confirming that the mutation does not prevent the interaction between p38α and TAB1.

### p38α(T185G) prevents TAB1-induced autophosphorylation without interfering with catalytic activity or preventing phosphorylation by upstream MAP2K.

**FIG 2:**
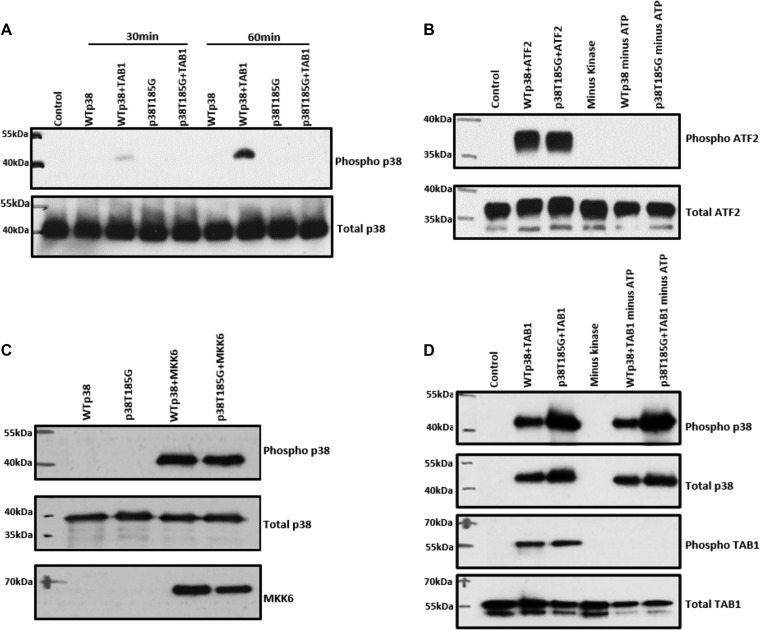
TAB1-mediated autoactivation is impaired in p38α(T185G) compared to wtp38α. (A) Western blot analysis of the products of an *in vitro* kinase assay performed with wtp38α or p38α(T185G) in the absence or in the presence of TAB1(384–412) peptide at 30 and 60 min. The T185G substitution impedes autoactivation. (C) Western blot analysis of *in vitro* activation of p38α(T185G) and wtp38α by upstream kinase MKK6*dd*. (B and D) Western blot analysis of an *in vitro* kinase assay of dually phosphorylated wtp38α or p38α(T185G) with ATF2 (B) and TAB1 (D), two known substrates of p38α. The mutant p38α(T185G) is catalytically competent.

**FIG 3:**
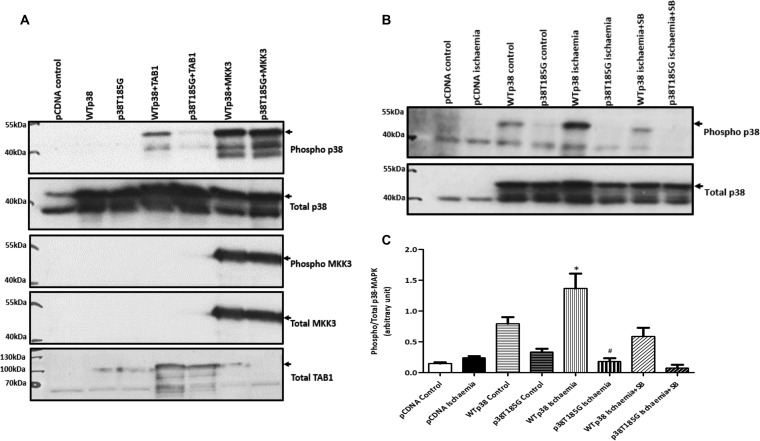
(A) HEK293 cells cotransfected with wtp38α or p38α(T185G) and TAB1 or MKK3. TAB1-mediated activation of p38α is impaired in the mutant, whereas no difference is detected between the wt and the T185G mutant with MKK3-mediated activation. (B) HEK293 cells transfected with wtp38α or p38α(T185G) exposed for 10 min to a buffer simulating ischemia. p38α activation is SB203580 sensitive, confirming autophosphorylation. Arrows indicate ectopic p38α, which is hemagglutinin tagged and heavier then endogenous p38α. (C) Quantification of phopho-p38α normalized against total p38α in HEK293 cells exposed to simulated ischemia (*n* = 3). *, *P* < 0.05 versus wtp38α control; #, *P* < 0.05 versus wtp38α ischemia.

To test our hypothesis, we carried out an *in vitro* kinase (IVK) assay using recombinant wtp38α or p38α(T185G) in the presence of TAB1(384–412). TAB1 caused autoactivation of wtp38α, whereas it was not able to cause autoactivation of p38α(T185G) (Fig. 2A). This result suggests that the hydrogen bond that forms between threonine 185 and aspartic acid 150 is essential in the process of p38α autoactivation caused by TAB1. To further examine this mechanism, we ectopically expressed p38α(T185G) in mammalian HEK293 cells together with TAB1. At 24 h after overexpression there was a clear increase in the phospho-p38α (T-G-Y) signal in cells transfected with wtp38α and TAB1; however, no such increment was observed in cells transfected with p38α(T185G) and TAB1 (Fig. 3A). These results in HEK293 cells recapitulate the result from the *in vitro* kinase assay and provide concrete evidence to support our hypothesis that the hydrogen bond formed between threonine 185 and aspartic acid 150 is a prerequisite for TAB1-induced p38α autoactivation.

Having obtained results in support of our hypothesis, we next examined whether hydrogen bond formation had a similar role in p38α's classical activation pathway. To investigate this, we carried out an *in vitro* kinase assay with p38α and the dual-specificity kinase MAP2K6, which is an upstream activator of p38. In an IVK reaction, the constitutively active MAP2K6*dd* was able to activate p38α(T185G) in a manner similar to that for wtp38α (Fig. 2C). We obtained the same result when we transfected HEK293 cells with the p38α(T185G) and MAP2K3 (Fig. 3A). MAP2K3 and MAP2K6 equally activated both wtp38α and p38α(T185G), suggesting that the classical activation pathway is not affected by the hydrogen bond between threonine 185 and aspartic acid 150. Next, we examined whether the catalytic activity of p38α(T185G) was affected. We carried out an *in vitro* kinase assay with active p38α(T185G) or active wtp38α and activating transcription factor 2 (ATF2) or the scaffold protein TAB1. ATF2 is a classic substrate of p38α, and TAB1 is both an activator of p38α through autophosphorylation and a substrate of p38α (16). The results from these IVK assays showed that p38α(T185G) phosphorylates ATF2 and TAB1 in a manner similar to that for wtp38α (Fig. 2B and D). These results suggest that the hydrogen bond between threonine 185 and aspartic acid 150 does not impact p38α's kinase activity toward its downstream substrates. They also reinforce the conclusions of the ITC experiment described in Table 1; namely, p38α(T185G) has an affinity toward TAB1 that is similar to that of wtp38α.

### p38α(T185G)-TAB1 complex crystal structure.

**FIG 4:**
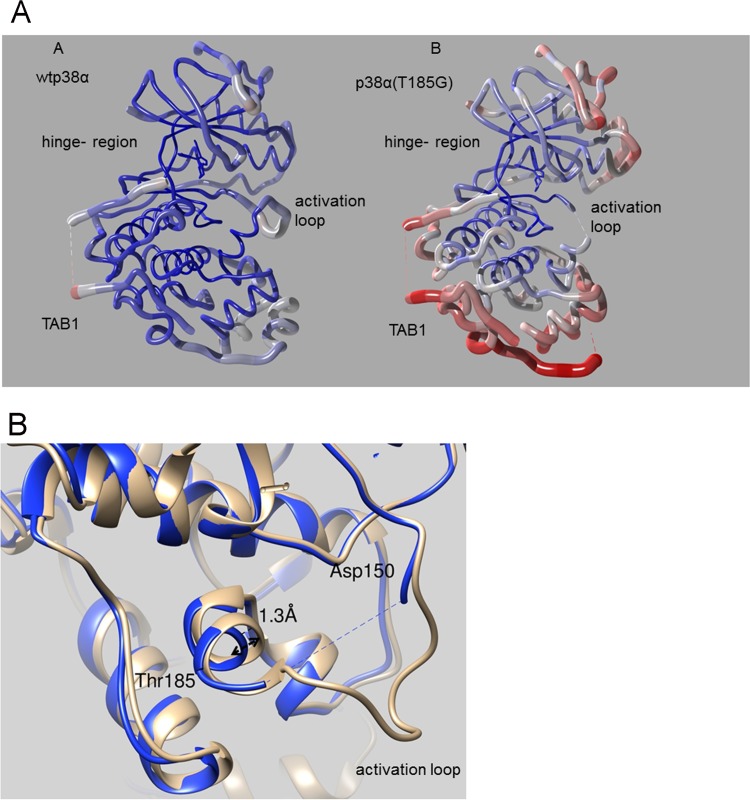
(A) Worm-format C-alpha trace representations of wtp38α-TAB1 (A) and p38α(T185G)-TAB1 (B) with worm radius and color temperature (blue [low] to red [high]) correlated to per-residue average B factor. Residues 173 to 181 of the activation loop are disordered (dashed line) in the T185G mutant and are omitted from the structure. The T185G mutation has caused an increase of the B factor values radiating out from the hinge region. (B) Superimposition of wtp38α-TAB1 (gray) and p38α(T185G)-TAB1 (blue) in ribbon representation, showing a zoom-in of the region containing residues 150 and 185. The helix containing residue 185 has relaxed away from Asp150 in the mutant structure (blue).

We then solved the X-ray structure of the p38α(T185G)-TAB1 complex (PDB code 5O90) (Table 2) and compared it with that of the wtp38α-TAB1 complex (PDB code 4LOO) (Fig. 4). As expected, the crystal structure revealed many features that are shared between these complexes. The mutation of p38α does not affect the TAB1 interaction, and in both structures TAB1 binds in a bipartite manner on the kinase C lobe and induces conformational changes that propagate through p38α: the N- and C-terminal lobes of p38α move toward each other, causing significant closure around the ATP-binding pocket. In the wt complex as part of this rearrangement, threonine 180 of the T-G-Y motif flips orientation so that it points into the active site, and residues Tyr182 to Thr185 of the activation loop form a short helical structure stabilized by a hydrogen bond between threonine 185 and aspartic acid 150; the remainder of the activation loop is also well ordered and clearly visible in the crystal structure. In contrast to this, in the T185G structure the majority of the activation loop is disordered and not visible (residues 173 to 181); the first turn of the helix from Tyr182 to Gly185 is still present as tyrosine 182 forms a crystal contact stabilizing this arrangement, but the helix has relaxed away from aspartic acid 150 and there is a substantial increase in the B factors of the helix residues (Fig. 4A).

**TABLE 2:** Statistics for the X-ray structure of the p38α(T185G)-TAB1 complex, PDB code 5O90

Parameter	Value for p38α(T185G)-TAB1^*a*^
Data collection	
Collection details	Diamond, IO2
Wavelength (Å)	0.9795
Space group	*P*2_1_
Unit cell dimensions	
*a*, *b*, *c* (Å)	45.9, 73.6, 59.0
α, β, γ (°)	90.0, 91.1, 90.0
Resolution (Å)	58.96–2.45
Completeness (%)	91.4 (87.0)
〈*I*/σ(*I*)〉	7.1 (0.9)
*R*_merge_ (%)	0.086 (0.637)
CC_1/2_ (%)	96.0 (41.3)
Multiplicity	1.9 (1.7)
Refinement	
No. of reflections	
Working set	13,808
Test set	617
*R*_work_/*R*_free_ (%)	24.9/27.5
Mean B factor (Å^2^)	87.7
Root mean square deviation	
Bond length (Å)	0.01
Angles (°)	1.18
Ramachandran statistics (%)	
Favored	95.6
Allowed	3.2
Outliers	1.2

a Values in parentheses represent the highest-resolution shell.

Moreover, the T185G mutation has had a profound impact on both the absolute B factor and B-factor distribution. As shown in Fig. 4A, the center of the T185G mutant complex close to the hinge region is still reasonably well ordered, but there is a strong correlation between distance from the hinge and increased average residue B factor, as opposed to the case for the wt structure. Consistent with these data, we propose that TAB1 in both structures causes the N and the C lobes of p38α to move toward each other, but the key hydrogen bond formed by threonine 185 and aspartic acid 150 is then required to lock the two domains together in the closed state. In the mutant structure, the absence of the hydrogen bond causes significant flexibility between the N- and C-terminal domains in the crystal structure, thus explaining the observed B-factor distribution; in addition, the T-G-Y motif is no longer oriented toward the ATP-binding site, thus impeding p38α autoactivation.

### p38α(T185G) inhibits TAB1-induced cell damage in HEK293 and H9C2 cells.

To further validate the selective inhibition of p38α, we transfected HEK293 cells with either wtp38α or p38α(T185G) and subjected them to simulated ischemia, an experimental setup that tries to recapitulate the low-oxygen and low-pH conditions experienced by cells during ischemia, and we relied on endogenous TAB1 to cause p38α activation. After 10 min of incubation in ischemic buffer, we detected increased phospho-p38α signal in cells transfected with wtp38α (Fig. 3B). This was inhibited by the ATP-competitive p38α inhibitor SB203580 (10 μM), confirming the mode of p38α activation to be autoactivation. In cells transfected with p38α(T185G), we observed a diminished phospho-p38 signal with ischemia, confirming impaired autoactivation.

**FIG 5:**
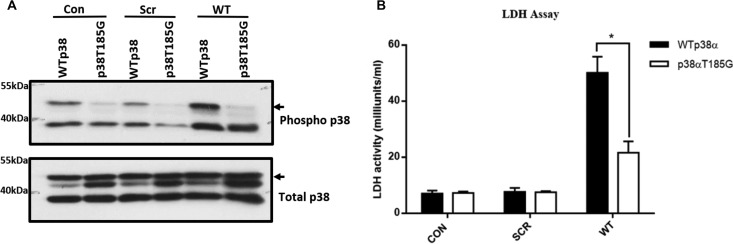
(A) LDH activity measured in culture medium collected from H9C2 cells expressing either wtp38α or p38α(T185G) that were incubated overnight with 20 μM scrambled peptide (Scr) or TAB1 peptide (WT). (B) Activation of p38α in H9C2 cells expressing either wtp38α or p38α(T185G) when exposed to 20 μM scrambled peptide (SCR) or TAB1 peptide (WT) overnight. Arrows indicate ectopic p38α, which is hemagglutinin tagged and heavier then endogenous p38α.

We next created a stable H9C2 myoblast cell line expressing either wtp38α or p38α(T185G). The cells were incubated with TAB1(384–412) peptide fused with a cell-permeative peptide derived from the HIV1 transactivator of transcription (TAT) overnight at 20 μM. The next day, the activity of lactate dehydrogenase (LDH), a marker of cell injury, was measured in the cell medium. The cells expressing wtp38α incubated with the TAB1 peptide produced a substantial increase in LDH activity compared to cells incubated with the scrambled form of TAB1 peptide. In cells expressing p38α(T185G), the TAB1 peptide incubation resulted in less LDH release than in the cells expressing wtp38α (Fig. 5A). The amount of LDH release correlated with the level of p38α activation (Fig. 5B). The result suggests that TAB1 causes cell injury via p38α activation, and the T185G mutation significantly diminishes the autophosphorylation of p38α and release of LDH.

## DISCUSSION

In this study, we have revealed a key structural change that occurs in p38α during TAB1-mediated p38α autoactivation. Previously we found that TAB1 binds to p38α in a bipartite manner, which causes a structural rearrangement within p38α that leads to its autoactivation (6). Here, we have built on this observation to show that the formation of a hydrogen bond between Thr185 and Asp150 is an important event in the autoactivation process. Using recombinant protein in IVK assays and exogenous protein expression in mammalian cells, we have shown that preventing the formation of this hydrogen bond significantly reduces p38α autoactivation and subsequent cellular damage. We have also solved the crystal structure of the p38α(T185G)-TAB1 complex to show that the hydrogen bond between Thr185 and Asp150 is crucial in initiating the autophosphorylation reaction, providing the energy to orient the T-G-Y motif toward the kinase catalytic site. In the mutant structure, as a result of the absence of the hydrogen bond between Gly185 and Asp150, the C-terminal end of the activation loop (Tyr182 to Thr185) has a higher temperature factor than in the wild type, the activation loop (Leu171 to Val181) is highly mobile to a point that it is not visible in the X-ray structure, and the T-G-Y motif is no longer locked next to the kinase catalytic site.

Every attempt to create a clinically effective small molecule to inhibit p38α has been unsuccessful. Despite many clinical trials with several drugs, none has passed the phase III stage, with losmapimod being the latest drug to fail (18). The major issue has been toxicity, such as liver damage, skins rashes, and gastrointestinal disorders, which is not surprising considering the ubiquitous nature of p38α expression and its nodal position in cell signaling (19, 20). Most p38α inhibitors are type I ATP-competitive inhibitors, which compete with ATP and inhibit p38's catalytic activity under all circumstances (21). Due to the high structural similarity of the ATP-binding sites, they nonselectively block alpha and beta isoforms of p38 and in some cases also the gamma and delta isoforms, depending upon their achieved concentration (22). This results in blanket inhibition of p38 under all circumstances, including during beneficial homeostatic function, thereby leading to harmful side effects. An alternative strategy is needed whereby we avoid blanket inhibition of p38 and aim to achieve circumstance-selective inhibition. This makes the TAB1-mediated p38α activation pathway an attractive target, since it may be a gateway to ischemia-selective inhibition. The data we have collected have unveiled a critical structural change in p38α during ischemia-triggered autoactivation induced via TAB1. Our findings reveal a mechanism that can selectively block p38α activation caused by TAB1 without affecting the classical activation pathway or other isoforms. This mechanism proves the concept that it is possible to selectively disable p38α catalytic activity and potentially achieve circumstance-selective inhibition of p38α during myocardial ischemia.

## MATERIALS AND METHODS

### Cloning and expression of p38α/p38α(T185G) in mammalian cells.

p38α(T185G) was cloned in two steps. First, the overlapping N-terminal and C-terminal regions were produced with complementary internal primers containing the desired mutations. In the second step, the PCR products from the first reaction were combined to form the template for a second PCR with external primers to produce a full-length p38α harboring the desired mutation. wtp38α, p38α(T185G), MKK3b, and TAB1 were cloned into pCDNA3 containing the cytomegalovirus (CMV) immediate early promoter and simian virus 40 (SV40) polyadenylation sequence for high-level expression in mammalian cells. wtp38α, p38α(T185G), and MKK3b had an N-terminal hemagglutinin (HA) tag, whereas TAB1 was CYP tagged. Gateway cloning technology was used to clone p38α/p38α(T185G) with a V5 tag into pCDNA6.2 that had the blasticidin resistance gene.

### Transfection.

HEK293 cells were seeded on 6-well plates in full growth medium (Dulbecco's modified Eagle's medium containing 584 mg/liter l-glutamine, 10% [vol/vol] fetal bovine serum, 1% [vol/vol] penicillin-streptomycin) at 37°C and transfected upon reaching 60% confluence. The transfection was done using Turbofect reagent. One hundred microliters of Opti-MEM solution was added to an Eppendorf tube, followed by 1 μg of DNA sample. Two microliters of Turbofect reagent was added to the tube, mixed gently, and left for 30 min for complex formation. An appropriate volume of Opti-MEM was added into the tube to make 1 ml total volume. The tube was inverted gently for complete mixture of the transfection reagents, which were then pipetted onto the cells in 6-well plates in a dropwise manner. The cells were incubated overnight at 37°C, after which the transfection medium was replaced with serum-free medium (SFM). In the experiments with inhibitors, drugs were incubated with SFM for the appropriate amount of time as needed. For Western blot analysis, the SFM was removed, cells were washed with phosphate-buffered saline (PBS), and 200 μl of 2× SDS sample buffer (120 mM Tris [pH 6.8], 6% [wt/vol] SDS, 20% [vol/vol] glycerol, 10% β-mercaptoethanol, and 0.01% bromophenol blue) was used to collect the cells. For stable transfection, H9C2 cells were transfected as described above but in the presence of blasticidin at 8 μg/ml. The cells were monitored over the next 2 weeks, replacing the medium and antibiotics every 3 days. At the end of 2 weeks, surviving cells stably expressing the protein were selected, transferred into a T75 flask, and grown normally under blasticidin selection at 8 μg/ml.

### Ischemia buffer and cell toxicity assay.

The recipe for the ischemia buffer was adapted from that described by Esumi et al. (23). Briefly, 50 ml of ischemic buffer (127 mM NaCl, 3.58 mM KCl, 0.49 mM MgCl_2_ · 6H_2_O, 1.8 mM CaCl_2_ · 2H_2_O, 4 mM HEPES, pH 7.4) was made up using 5 ml of 10× basic stock solution and 45 ml of sterile deionized H_2_O; 10 mM 2-deoxy-d-glucose (2-DG) and 20 mM Na lactate were added to the 50-ml solution, filter sterilized using a 0.2-μm syringe, and warmed to 37°C (the pH of the solution at this point is 6.8). Once the solution was warmed, 1 mM Na dithionite was added to the solution (the pH drops to 6.3) and mixed before quickly adding it onto the cells. The cells were then incubated in room air supplemented with 5% CO_2_ in a water-saturated incubator at 37°C for 10 min. After 10 min, cells were collected using 200 μl of 2× SDS buffer and analyzed by Western blotting. To measure the cell toxicity caused by TAB1 peptide, an LDH assay kit (MAK066; Sigma) was used. H9C2 cells were incubated with 20 μM TAB1 peptide in SFM overnight, and the cell medium was used to carry out the LDH test. The TAT-TAB1(384–412) peptide was purchased from Activotech. The TAB1 sequences are as follows: TAT-TAB1 (wt), GYGRKKRRQRRRGEMSQPTPTPAPGGRVYPVSVPYSSAQSTSKTSVTLSLVMPSQGQMV; TAT-TAB1 (scrambled), GYGRKKRRQRRRGTVSQGPASATPAYPPGGRVESQPLPSQSTLGAPSKTSVTSAVQSMM.

### Expression and purification of p38α/p38α(T185G) in Escherichia coli.

p38α and p38α(T185G) were cloned in the first multiple-cloning site of the pETDuet vector. The p38α gene was cloned with a His_6_ tag followed by a TEV cleavage site at the N-terminal end. The DNA was transformed in E. coli strain Rosetta II cells (Novagen), and ampicillin was used for selection. The protein expression and purification were carried out as described in reference 24.

### ITC.

Isothermal titration calorimetry (ITC) was carried out in an ITC200 microcalorimeter from Microcal (GE Healthcare Life Sciences). Microcal Origin 7.0 data software was used to analyze the result. Integrated heat data obtained for the titrations corrected for heats of dilution were fitted with a nonlinear least-squares minimization algorithm to a theoretical titration curve. The data fitting gave us the values for binding stoichiometry (*n*), binding affinity (*K*), and molar enthalpy change (Δ*H*). The molar entropy (Δ*S*) was calculated using Δ*S* = Δ*H* − ΔG, where Δ*G* = −*RT* × ln *K* (*R* = 1.987 cal mol^−1^ *K*^−1^ and *T* = 298 K).

### Crystallization and structure determination.

The p38α(T185G)-TAB1 complex at 12 mg/ml was preincubated with 1 mM SB203580 and crystallized by sitting-drop vapor diffusion at 4°C. The complex crystals grew in 25% (wt/vol) medium-molecular-weight polyethylene glycol (PEG) smears, 0.2 M ammonium sulfate, 0.01 M CdCl_2_, and 0.1 M HEPES, pH 7.5. The crystals were cryoprotected with mother liquor supplemented with 20% ethylene glycol. The diffraction data were collected using beamline I02 at Diamond Light Source and processed and scaled with MOSFLM and SCALA. The structure was solved by molecular replacement with a p38α monomer and PHASER as a search model.

### IVK assay.

An *in vitro* kinase (IVK) assay was performed to activate p38α by TAB1(384–412), p38α by MKK6, and ATF2 and TAB1 by p38α. p38α (3 μM) was activated with 15 μM TAB1, 3 μM p38α was activated with 0.6 μM MKK6*dd*, and 0.86 μM ATF2 or TAB1 was activated with 0.2 μM p38α. The reaction was carried out in 1× kinase buffer (25 mM Tris-HCl [pH 7.5], 5 mM β-glycerolphosphate, 2 mM dithiothreitol [DTT], 0.1 mM Na_3_VO_4_, and 1 mM MgCl_2_). ATP (550 μM) was added to the incubation mixture to start the reaction at 37°C for 2 h. At the end of the reaction, samples were collected with 2× sample buffer (20% glycerol, 6% SDS in 0.12 M Tris [pH 6.8], 10% 2-mercaptoethanol, and 0.4% bromophenol blue), heated to 95°C for 10 min, and run on 10% SDS gel under denaturing conditions. The samples were transferred onto a polyvinylidene difluoride (PVDF) membrane using semidry technique, blocked with 4% milk plus 1% bovine serum albumin (BSA) for 1 h at room temperature, and probed with an appropriate primary antibody overnight at 4°C. The blots were probed with an appropriate secondary antibody linked with horseradish peroxidase (HRP) and visualized by enhanced chemiluminescence. The antibodies used included anti-dually phosphorylated p38 (Thr180/Tyr182) (M8177; [Sigma] and 9211; Cell Signaling Technology [CST]) at 1:5,000, total p38 (9212; CST) at 1:10,000, anti-phosphorylated TAB1 (Phil Cohen group, Dundee) at 1:2,000, total TAB1 (Phil Cohen group, Dundee) at 1:4,000, anti-phosphorylated ATF2 (9221; CST) at 1:4,000, total ATF2 (20F1; CST) at 1:8,000, and anti-phosphorylated MKK3/6 (9231; CST) at 1:1,000.

### Accession number(s).

The X-ray structure of the p38α(T185G)-TAB1 complex has been deposited under PDB code 5O90.
